# Environmental Properties and Applications of Biodegradable Starch-Based Nanocomposites

**DOI:** 10.3390/polym14214578

**Published:** 2022-10-28

**Authors:** Ashoka Gamage, Punniamoorthy Thiviya, Sudhagar Mani, Prabaharan Graceraj Ponnusamy, Asanga Manamperi, Philippe Evon, Othmane Merah, Terrence Madhujith

**Affiliations:** 1 Chemical and Process Engineering, Faculty of Engineering, University of Peradeniya, Peradeniya 20400, Sri Lanka; 2 Postgraduate Institute of Agriculture, University of Peradeniya, Peradeniya 20400, Sri Lanka; 3 School of Chemical, Materials and Biomedical Engineering, University of Georgia, Athens, GA 30602, USA; 4 Department of Chemical Engineering, College of Engineering, Kettering University, Flint, MI 48504-6214, USA; 5 Laboratoire de Chimie Agro-Industrielle (LCA), Institut National de la Recherche Agronomique, Université de Toulouse, CEDEX 4, 31030 Toulouse, France; 6 Département Génie Biologique, IUT A, Université Paul Sabatier, 32000 Auch, France; 7 Department of Food Science and Technology, Faculty of Agriculture, University of Peradeniya, Peradeniya 20400, Sri Lanka

**Keywords:** biodegradability, carbon nanotubes, graphene, life cycle analysis, nanocomposites, packaging, remediation, starch

## Abstract

In recent years, the demand for environmental sustainability has caused a great interest in finding novel polymer materials from natural resources that are both biodegradable and eco-friendly. Natural biodegradable polymers can displace the usage of petroleum-based synthetic polymers due to their renewability, low toxicity, low costs, biocompatibility, and biodegradability. The development of novel starch-based bionanocomposites with improved properties has drawn specific attention recently in many applications, including food, agriculture, packaging, environmental remediation, textile, cosmetic, pharmaceutical, and biomedical fields. This paper discusses starch-based nanocomposites, mainly with nanocellulose, chitin nanoparticles, nanoclay, and carbon-based materials, and their applications in the agriculture, packaging, biomedical, and environment fields. This paper also focused on the lifecycle analysis and degradation of various starch-based nanocomposites.

## 1. Introduction

In recent days, nanocomposites have gained much attention over traditional composite materials and are widely used in food, packaging, biomedical applications, electronics, energy storage, optics, the automotive industry, bio-sorbants for environmental remediation, textiles, and many other applications [1,2]. Polymer nanocomposites consist of polymer matrices embedded with nanofillers [3]. Petroleum-based polymers are produced in huge amounts globally. Petroleum-based polymers are non-biodegradable, non-renewable, and produce hazardous substances which can threaten human health and the environment [4]. Furthermore, the depletion of these non-renewable petroleum-based fuels demands alternative resources [5].

Thus, biopolymer-based nanocomposites can be a sustainable alternative for petroleum-based nanocomposites in many applications due to their biodegradability, eco-friendliness, renewability, relatively inexpensive, low toxicity, abundancy, and improved thermal, mechanical, physical, barrier, and functional properties [3,4]. Various natural biopolymers, including starch, cellulose, pectin, lignin, chitin/chitosan, alginates, hyaluronic acid, gelatin, terpenes, gelatin, gluten, and polyhydroxyalkanoates (PHAs) from plants, animals, algae, microorganisms and synthetic biopolymers, including polycaprolactone (PCL), poly(butylene succinate) (PBS), poly(lactic-co-glycolic acids) (PLGA), and polylactic acids (PLA), have been used in nanocomposite materials for various applications [1,2,3,6,7,8].

Starch is one of the most abundant natural polymers globally. Starch and its nanocomposites have been extensively studied for their abundance, low cost, ease of processibility, and chemical and physical properties [1,4]. Furthermore, starch can be used in natural or modified form. Native starch has drawbacks, such as poor mechanical properties, high hydrophilicity, and high biodegradability. Thus, researchers are exploring starch modification techniques to improve its properties and develop novel composites [1].

Starch can be modified into nanoparticles and can also undergo various physical (milling, blending with other polymers, extrusion, plasticizers, etc.) and chemical (substitution, graft co-polymerization, cross-linking, oxidation, etherification, esterification, dual modification, etc.) modifications to produce materials with novel properties [9,10,11,12].

Starch can be reinforced with starch nanoparticle/starch nanocrystals and nano polymers such as nanoclay (montmorillonites [MMTs], halloysites nanotubes [HNTs]), carbon nanotubes (CNTs), and nanofibers and nanowhiskers (cellulose, chitin) and metal and metal oxides (TiO_2_ NPs, ZnO NPs, etc.) to achieve desirable properties and produce potential green sustainable nanocomposite materials [4,7,13]. The addition of nanofillers and additives with antioxidant and antimicrobial properties has been shown to improve or minimally affect biodegradation of starch-based nanocomposites [5,14,15]. Lifecycle assessments on starch and starch-based composites ensure their lower environmental impact and sustainable alternative for petrochemical-based polymers [16,17,18].

This review mainly discusses the starch-based nanocomposites in regard to starch and its nanostructures, various starch-based nanocomposites mainly reinforced with nano polymers, such as nanoclay, carbon-based materials, nanocellulose, and chitin NPs), and their applications, particularly in the fields of agriculture, packaging, biomedicine, and the environment. Moreover, this paper highlights the lifecycle analysis and degradation of various starch-based nanocomposites in order to analyze their environmental impact.

## 2. Starch

**Figure 1:**
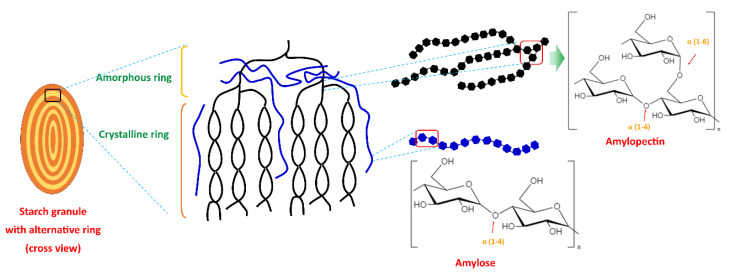
Starch granule structure and the chemical structure of amylopectin and amylose.

Starch is a polysaccharide and is renewable, inexpensive, biodegradable, and readily available. Starch contains two polymers (glucans) known as amylose (10–30%) and amylopectin (70–90%). Amylose is a linear chain of D-glucose units linked by the α-(1,4) glycosylic bonds, while amylopectin is a highly branched and high molecular weight chain composed of D-glucose repeating units linked by α-(1,4) glycosylic bonds and α-(1,6) glycosidic bonds. The amylopectin chain contains 10–60 glucose units, and the side chains consist of 15–45 glucose units with about 5% of α-(1,6) branching points [6,7]. Amylose and amylopectin are radially arranged in an alternating concentric (amorphous and semi-crystalline) ring in starch granules. Amylopectin is radially arranged in granules and contributes to its crystalline nature (double helices region), and single helices amylose is randomly distributed among amylopectin clusters. Amylose and the branching point of amylopectin form the amorphous region [19,20,21]. Figure 1 illustrates the structure of the starch granule and the chemical structure of amylopectin and amylose.

Starch is a primary energy source in plants, which is stored in various parts, including the roots, tubers, seeds, and stems [6]. Various plant sources, such as corn, potato, wheat, cassava, rice, corn, barley, rye, millet, peas, mung beans, lentils, arrowroot, sago, sorghum, banana, yam, and many others, are utilized to obtain starch [22,23,24].

Starches from different sources show variation in their chemical composition (α-glucans, moisture, lipids, proteins, and phosphorylated residues), the structure of glucan components (amylose and amylose), and starch granule size and shape due to genetic and environmental factors [25,26].

Starch granules’ size and shape can vary with the content, structure, and arrangement of amylose and amylopectin [25]. Starch granules are found in various sizes ranging from 2–150 µm and packed with amylose and amylopectin content. Regular starch granules contain amylose in the range of 15–30% but can be varied in the range of 0–78%. Waxy starch contains lower or no amylose, whereas high-amylose starch consists of more than 50% amylose [7,23]. Table 1 shows the amylose contents of various starch sources.

Starch-based hydrogel is formed via gelatinization of starch during heating with excess water and followed by three-dimensional network formation by retrogradation [37]. Gelatinization of starch is an irreversible process that occurs through the absorption of water and disruption of the crystalline structure of starch granules by hydrogen bond breakage, swelling, the disintegration of starch granules, leaching of amylose that increases viscosity and solubilization of starch molecules [32,35,37].

Amylose and amylopectin content, amylopectin structure (molar mass or chain length), and starch granule size influence the chemical, physical, optical/transparency, and functional properties (water uptake, swelling, gelatinization, pasting [pasting viscosity and temperature], retrogradation, and susceptibility to enzymatic hydrolysis of starch [7,20,23,36,38].

Amylopectin contributes to water absorption, swelling, and pasting of starch granules, whereas amylose hinders the swelling property in the presence of lipids, thus preventing gelatinization power [32,38]. Furthermore, short-chain amylopectin showed better swelling power than that of long-chain amylopectin, indicating that starch with higher crystallinity reduces the swelling power [38]. Smaller granule size increases hydration, thus increasing the swelling, viscosity, and gelatinization properties [26].

Amylose content is negatively correlated with swelling power, gelatinization temperature, and the enthalpy of gelatinization required to disrupt the crystalline structure [35]. Waxy starch has a higher degree of crystallinity and higher gelatinization temperature than starch with high amylose content [31,35]. Amylose in starch has a high tendency for retrogradation due to its linear structure. However, the retrogradation properties of starch are mainly determined by the degree of crystallinity and gelatinization temperature than the amylose content [35].

Amylose–amylopectin ratio also influences thermal, mechanical, and barrier properties. Basiak et al. [23] reported that potato starch, containing lower amylose (20%) than that of wheat (25%) and corn (27%) starch, exhibited greater mechanical properties and lower water solubility, water vapor, and oxygen permeability. Other than that, optical properties were influenced by the amylose/amylopectin ratio: the potato (lower amylose) film was transparent, whereas corn and wheat films were opalescent.

However, applications of starch have been limited due to their poor performance, such as through their brittleness, high water sensitivity, poor gas and moisture barrier, susceptibility to retrogradation, high viscosity, and limited solubility [13,39]. Therefore, plasticizers, chemical modifiers, and incorporating nanofillers, such as starch nanoparticles, nanoparticles, nanoclay, nanofibers, and others, have been used to improve the properties of starch [39].

## 3. Nanomaterials and Nanocomposites

**Figure 2:**
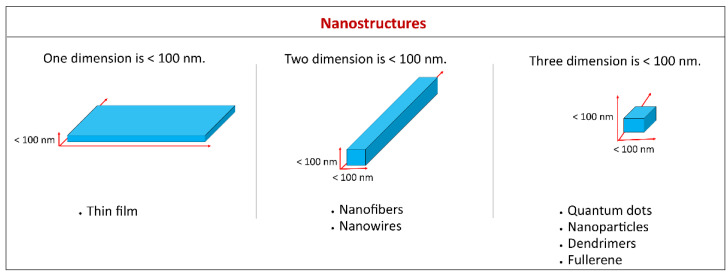
Examples of various types of nanomaterials based on the number of dimensions in the nanometer range.

Nanomaterials are referred to as materials which have at least one of their dimensions less than 100 nm. Based on the definition, a thin film with <100 nm thickness is a nanomaterial as one of the dimensions is nanometric. Likewise, nanomaterials such as nanofibers, nanowires, and nanorods have two dimensions on the nanoscale, whereas quantum dots, nanoparticles, dendrimers, and fullerene have three dimensions in the nanometer range (Figure 2) [40].

**Figure 3:**
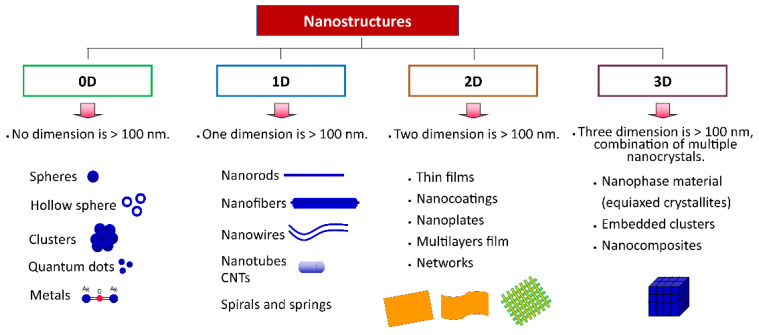
Classification of materials based on dimensionality.

Nanomaterials can be classified based on dimensionality (number of dimensions with a length larger than 100 nm), as shown in Figure 3: 0D, 1D, 2D, and 3D. Zero dimension (0D), including spheres, hollow spheres, clusters, quantum dots, and metals, have no dimension of particles larger than 100 nm, i.e., all dimensions in the nanoscale. One-dimensional (1D) nanomaterials, such as nanorods, nanowires, nanofibers, and nanotubes, have one dimension, not in the nanoscale (>100 nm) and the other two are in the nanoscale, whereas two-dimensional (2D) nanomaterials, including thin film, nanocoatings, nanoplates, and nanolayers, have two dimensions, not in nanoscale and another one in nanoscale. Three-dimensional (3D) is the combination of nanocrystals in different directions which have various dimensions above 100 nm. Figure 3 depicts the classification of nanomaterials based on dimensionality [40,41,42].

**Figure 4:**
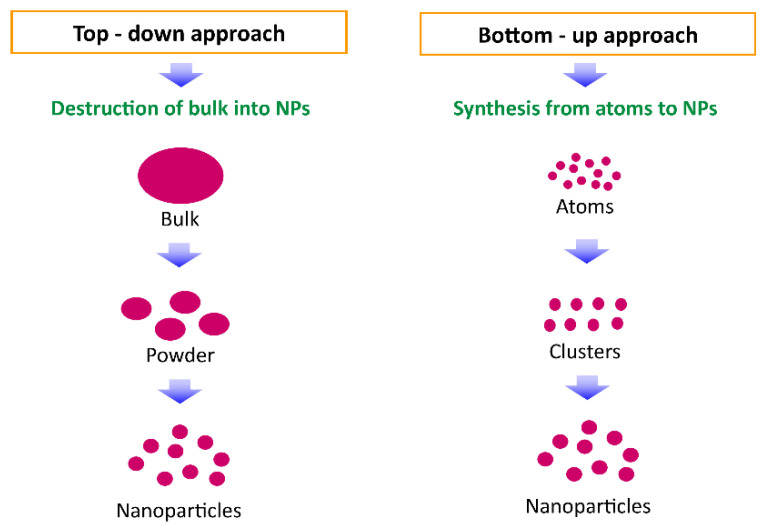
Nanoparticle synthesis methods: top-down and bottom-up approach.

Nanomaterials can be synthesized by two approaches: top-down and bottom-up approaches (Figure 4). In the top-down method, the bulk material is restructured into nanomaterials using mechanical grinding/milling, ball milling, polishing, lithography, and other means. While in the bottom-up method, nanomaterials are assembled from atomic range particles/molecules or nanoclusters through the sol–gel method, spinning, molecular self-assembly, pyrolysis and condensation, vapor phase deposition, and other methods [40,41].

Composite materials consist of two or more dissimilar materials, which are composed of two major constituents: (1) a matrix as a continuous phase (polymer, ceramic, or metal) and (2) reinforcement materials as an un-continuous phase. Bionanocomposites are composite materials that are composed of biopolymers and particles with at least one dimension in the nanometer range (1–100 nm). Bionanocomposites can also be referred to as green composites or biohybrids, or bioplastics [3,43].

**Figure 5:**
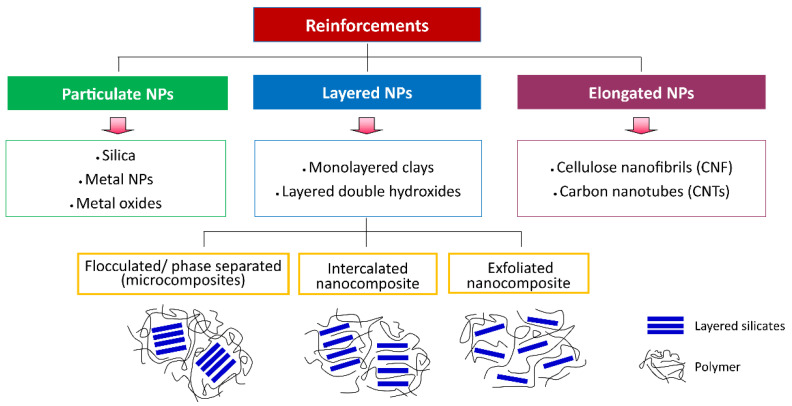
Classification of the nanocomposites.

Nanocomposites can be classified into three categories based on the morphology of reinforced nanoparticles: (1) particulate/iso-dimensional (silica, metal NPs, metal oxides), (2) layered (monolayered clays, layered double hydroxides), and (3) elongated (cellulose nanofibrils [CNF], carbon nanotubes [CNTs]) nanoparticles [3,44]. Particulate reinforcements are used to enhance resistance to flammability and reduce permeability and cost, whereas layered reinforcements are used for their superior mechanical behavior [43]. Furthermore, based on the degree of dispersion of particles in the matrix, layered nanocomposites have three subclasses, including intercalated, exfoliated, and flocculated/phase-separated nanocomposites (micro-composites) [3,6,43]. Flocculated/phase-separated nanocomposites are formed without a partition between individual layers due to the particle–particle interactions, polymer chains are intercalated between sheets of layered nanoparticles in intercalated nanocomposites, and exfoliated nanocomposites are formed by partition between individual layers (Figure 5) [43].

## 4. Starch Nanoparticles (SNPs)

Starch nanoparticles (SNPs) are mainly synthesized by the methods of hydrolysis (acid or enzymatic), regeneration, and physical treatments (milling, high-pressure homogenization, gamma radiation, and ultra-sonication) [45].

SNPs are mainly used as fillers in a polymer matrix to improve their reinforcing effect and mechanical and barrier properties [13]. Nanoparticles have a large surface area/volume ratio, allowing a great interaction capacity, which makes them potential reinforcement materials [46]. SNPs are non-toxic and can be used to prepare nanocomposite, absorbent, carrier (encapsulation), and emulsion stabilizers for food and non-food applications [45,47,48].

Santana et al. [46] reported the SNP obtained from ultrasound showed a significantly higher yield than SNP synthesized by acid hydrolysis. In addition, incorporating SNPs reduced the water vapor permeability of starch film [46]. Lin et al. [49] prepared debranched starch nanoparticles (DSNPs) by reverse emulsification using debranched waxy corn starch (98% of amylopectin), which showed a higher crystallinity and melting temperature than that of native waxy corn starch. Furthermore, the addition of debranched starch nanoparticles (5 wt.%) into corn starch films improved the tensile strength by 85.9% and decreased water vapor permeability and the oxygen transmission rate by 30.94% and 79.31%, respectively.

In another study, starch NPs prepared by acid hydrolysis containing Ag NPs showed good antibacterial activity against *Staphylococcus aureus*, *Salmonella typhi*, and *Escherichia coli* which has the potential to be used as a coating material for food packaging [50].

## 5. Starch-Based Nanocomposites

Native starch or thermoplastic starch (TPS) has poor mechanical properties (fragility/brittleness), low thermal stability, hydrophilicity, high water vapor permeation, poor resistance to external factors (humidity, tearing, picking, etc.), and a lack of compatibility with hydrophobic polymers [7,12,51]. Therefore, starch is blended with other natural and synthetic polymers or incorporated with various nanomaterials to enhance the physical, mechanical, and barrier properties [7]. Compared with bulk materials, nanoparticles have a surface area/volume ratio and possess unique physical, mechanical, optical, magnetic, electrical, and other properties [42]. Hence, recently, bionanocomposites can be a promising material to enhance mechanical and barrier properties [52]. Starch reinforced with nanofillers, including nanocellulose, chitin nanoparticle, nanoclay, and carbon-based materials, are discussed below.

### 5.1. Starch/Nanocellulose Composite

**Figure 6:**
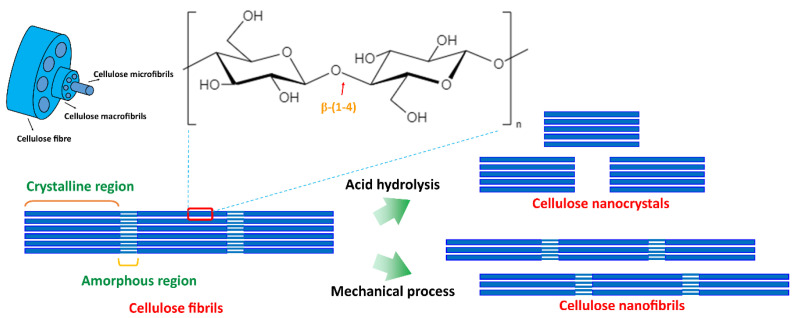
Cellulose chemical structure and schematic diagram of the formation of cellulose nanocrystals and cellulose nanofibrils.

Cellulose is the primary component of the plant cell wall and can be extracted from plants, invertebrates, marine animals, algae, fungi, and bacteria [53]. It is the most abundant natural polymer and is popular for its mechanical properties, reinforcement capabilities, low density, renewability, low toxicity, and biodegradability [54]. Cellulose is the polymer of D-glucose units linked by β-(1,4)-glycosidic bonds, and higher hydroxyl groups (-OH and -CH_2_-OH) at equatorial positions give higher stability (Figure 6) [55]. Cellulose fibres are formed with strong inter and intramolecular hydrogen bonds and aggregate with highly ordered (crystalline) and disordered regions (amorphous) [56]. Nanocellulose is a nanostructure of cellulose and has drawn much attention over the past years due to its excellent characteristics, including its high aspect ratio (length to diameter), improved mechanical and thermal properties, crystallinity, flexibility, renewability, abundance, biocompatibility, and biodegradability [55,57].

**Figure 7:**
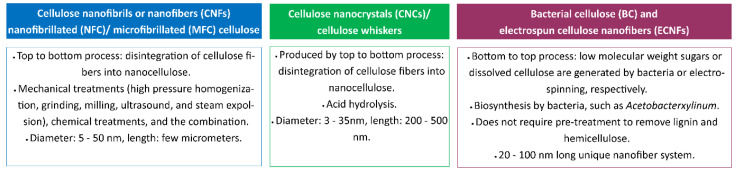
Types of nanocelluloses.

Nanocellulose can be produced by top-down and bottom-down processes (Figure 6) [53,54] using various techniques, including enzymatic techniques, chemical hydrolysis, and mechanical treatments, including high-pressure homogenization, grinding, cryo-crushing, micro-fluidization, and high-intensity ultrasonication [46,53,54]. These synthetic techniques and conditions influence the dimensions, composition, and properties of nanocellulose. Nanocellulose can be generated in three forms: (1) cellulose nanofibrils (CNFs) and (2) cellulose nanocrystals (CNCs) from woods and other lignocellulosic materials using a top-down process, and (3) bacterial cellulose (BC) from the biosynthesis of bacteria using a bottom-to-top process. Figure 7 summarizes the three forms of cellulose and synthesis methods [53,55].

Nanocellulose is widely used in various applications, such as biomedical engineering, the automotive industry, electronics, food packaging, cosmetics, construction, textiles, wood adhesives, and wastewater treatment applications [53,57].

Othman et al. [58] prepared the corn starch (CS) film reinforced with nanocellulose fiber (NCF) and thymol, a compound extracted from the essential oil of thyme, which has antioxidant and antimicrobial properties. They reported that adding 1.5% of NCF improved the thermal stability, mechanical, and barrier (water vapor and oxygen) properties of corn starch film. The CS/NCF/thymol composite reported improved thermal stability and flexibility. However, a significant reduction was observed with tensile strength, Young’s modulus, and barrier properties [58]. In another study, starch from an unripe plantain bananas reinforced with cellulose nanofibers from banana peels improved the mechanical and water vapor barrier properties [59]. Starch/CNC nanocomposites were reported to improve the tensile strength (2.8 to 17.4 MPa), Young’s modulus (112 to 520 MPa), and water barrier properties, as well as reduce the water solubility (26.6 to 18.5%) and contact angle 38.2 to 96.3° [60].

### 5.2. Starch/Chitin Nanoparticles Composites

**Figure 8:**
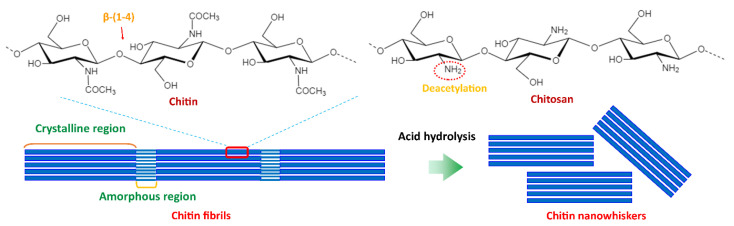
Chemical structure of chitin and schematic diagram of the formation of chitin nanowhiskers.

Chitin is the second most abundant natural polysaccharide next to cellulose and is found in the shell of crustaceans (crab, lobster, and shrimp), the exoskeleton of arthropods, molluscan shells of squid, mushrooms, the cell wall of algae and fungi (yeast and mold). Chitin is composed of N-acetyl-2-amido-2-deoxy-β-D-glucose (N-acetylglucosamine) units linked with a β-(1,4)-glycosidic bond, in which acetamide groups (−NHCOCH3) consists at the C2 of cellulose monomer. Chitosan is derived from the alkaline deacetylation of chitin (Figure 8). Chitin crystals are found in three forms: α-chitins (which contain antiparallel cellulose chains), β-chitins (parallel cellulose chains), and γ-chitin (among three chains, two of them are in the same direction, and one is in the opposite direction) [61,62,63].

Chitin nanomaterial can be prepared through top-down and bottom-up approaches. Chitin fibrils consist of amorphous and crystalline regions and thus can be converted into three types of nano-chitins in a top-down approach: nanocrystals (via acid hydrolysis), nanofibers (via mechanical treatments), and nanowhiskers (consecutive acid hydrolysis at a high temperature and mechanical treatments) [39,62].

Nano-chitin has been widely studied for its high aspect ratio, high surface area, good mechanical properties, lightweight/low density, good chemical stability, renewability, non-toxicity, and antibacterial properties, and it is used in biomedicine, packaging, water treatment, green electronics, cosmetics, and many other applications [61,63].

A combination of chitin nanofibers and starch nanoparticles showed higher emulsion stability over a range of pHs and temperatures and can be used as an emulsion stabilizer in various products, such as food, paint, coating, cosmetics, and pharmaceuticals [48].

Chang et al. [64] reported chitin nanoparticles (CNPs) exhibited lower crystallinity than chitin whiskers. At a low level of CNPs, tensile strength, storage modulus, glass transition temperature, and water vapor barrier properties of plasticized potato starch/CNPs nanocomposite due to good interfacial interaction between CNPs’ nanofiller and starch matrix.

By adding 5 wt.% chitin nanofibers (CNF) obtained from the fungus *Mucor indicus*, Young’s modulus and the tensile strength of TPS were enhanced by 239% and 216%, respectively, and moisture absorption was reduced from 51% to 38%. However, the addition of CNF at a higher level increased moisture absorption and reduced the mechanical properties of TPS [39]. In another study, Heidari et al. [61] reported that CNF/TPS nanocomposite films were more permeable to water vapor than pure CNF film. CNF at higher levels lowers the dispersion of nanofiller and tends to agglomerate, which leads to poor water vapor barrier and mechanical properties. In addition to that, the presence of excessive NH_2_ groups at the CNF surface may increase the affinity to water, thereby increasing water absorption [39,61].

### 5.3. Starch/Nanoclay Nanocomposites

Clay is a polymer composite of two-dimensional layered mineral silicates. The single layer is formed by the edge-linked octahedral sheet of aluminum or magnesium oxide sandwiched between two tetrahedral silicate sheets. As shown in Figure 6, three types of polymer-based nanocomposites can be obtained based on the polymer and silicate layers. Silicate clay is characterized by important physical properties, such as a cation exchange capacity and specific surface area [65,66]. Polymer/nanoclay composites are used in the automotive industry, aeronautical industry, packaging, flame-resistant materials, biomedical applications, and wastewater treatment [67,68]. Nanoclays can be categorized into several classes: smectite, chlorite, kaolinite, illite, and halloysite [68].

Plate-like montmorillonite (MMT) (smectite), a multilayer-aluminosilicates, has been widely studied as a reinforcing material in polymers due to its excellent cation exchange capacity, swelling behavior, and large surface area [68,69]. MMT also improved the thermal stability, mechanical, optical, and barrier properties, even at their lower concentration [70].

Mohan et al. [15] reported that the incorporation of MMT nanoclay into corn starch-based film resulted in a significant reduction in water absorption (by 22%), moisture uptake (40%), oxygen permeation (30%), and swelling thickness (31%) in comparison to corn starch film. Furthermore, the concentration of MMT nanoclay determines the structure of the nanocomposite. X-ray diffraction (XRD) analysis revealed that the intercalated nanoclay structure forms at a higher concentration (>2%), whereas the exfoliated structure forms at a lower concentration in the polymer matrix [15]. In another study, MMT addition was also shown to improve the tensile strength and biodegradability in cross-linked PLA/maleated TPS nanocomposite [71]. Biodegradable nanocomposites fabricated from cross-linked wheat starch (CLWS)/sodium montmorillonite (Na-MMT)/TiO_2_ NPs showed an exfoliated structure. Incorporating Na-MMT and TiO_2_ NPs reduced the water vapor permeability and water solubility of the CLWS film, whereas thermal stability, tensile strength, and Young’s modulus were increased. TiO_2_ NPs showed better UV-blocking properties than Na-MMT [69]. Maize starch/glycerol (20%)/Na-MMT (10%) nanocomposite also showed intercalated structures and improved tensile properties [66].

Iamareerat et al. [72] prepared nanocomposite film with plasticized cassava starch incorporated with sodium-bentonite and cinnamon essential oil. The addition of sodium-bentonite nanoclay (0.5–0.75%) decreased the water vapor permeability in plasticized cassava starch with 2% glycerol film. Further addition of cinnamon essential oil into the CS/glycerol (2%)/sodium-bentonite (0.75%) showed better antibacterial activity and significantly inhibited microbial growth in pork meatballs, despite the increase in water vapor permeability.

Halloysites clay nanotubes (HNTs), aluminosilicate hollow cylinders, have a lower hydroxyl group on the surface than other silicates such as MMT, making them a promising reinforcement material for polymers [68,73]. Furthermore, HNTs exhibit exfoliated structures due to their high aspect ratio [73]. Dang et al. [73] revealed that the addition of modified or unmodified HNTs into the TPS/poly(butylene adipate-co-terephthalate) (PBAT) blend improved the thermal and mechanical properties without loss of ductility of the plasticized wheat starch matrix [74]. Another investigation on PVA/starch/glycerol/HNTs nanocomposites revealed that their hydrophobic nature and biodegradability decreased with the addition of HNTs [75].

### 5.4. Starch/Carbonaceous Nanocomposites

Fullerenes, diamonds, carbon nanotubes (CNTs), graphene, and their derivatives are common carbon allotropes used in carbon-based nanocomposites [76].

CNTs found in two forms, single-walled (SWCNT) or multi-walled carbon nanotubes (MWCNT), have been widely studied as reinforcing fillers for TPS nanocomposite films [77]. CNTs have a larger surface area, excellent electrical conductivity, mechanical and thermal properties and they also have a higher volume-to-area ratio compared to that of other nanoparticles and they are widely used in various biomedical applications, environmental pollution control, sensing and detection, the automobile industry, and secondary food packaging. Direct contact food packaging materials are limited by their migration and potential toxicity [76,78,79].

Electrically conductive biocomposite films have gained popularity in various electronic, biomedical, and food packaging applications [22]. Potato starch-based film reinforced with MWCNT and ionic surfactants (sodium cholate, SC; cetyltrimethylammonium bromide, CTAB) decreased the contact angle and showed improved antioxidant properties (30.2 and 12% of scavenging activity, respectively) due to the presence of MWCNT. Surfactant SC showed better dispersibility of MWCNT in a potato starch matrix with improved mechanical properties and crystallinity [22].

Starch plasticized with ionic liquids reduces the retrogradation resulting in increased film stability and it has the potential use in ionically conducting solid polymers. The addition of nanofiller MWCNT at 0.5 wt.% in starch plasticized with ionic liquid, 1-ethyl-3-methylimidazolium acetate ([emim+][Ac−]) significantly increased the tensile strength by 327%, Young’s modulus by 2484%, and elongation at break 82% (from 30 to 69%). Moreover, electrical conductivity was increased with MWCNT content (wt.%) and reached a maximum (56.3 S/m) at 5 wt.% MWCNT. MWCNT/starch plasticized with [emim+][Ac−] showed electroconductive properties because of its ionic nature of ionic liquids and the excellent electrical conductivity of MWCNT [77]. A starch–iodine complex matrix reinforced with a small amount of MWCNT (0.055%) reduced the water vapor permeability by 43% [78].

Graphene is a two-dimensional material arranged in a hexagonal lattice. Plasticized starch incorporated with reduced graphene oxide (rGO), a derivative of graphene, exhibited increased conductivity and dielectric properties, which could make it a potential candidate for producing sustainable bio-friendly electronic devices [80].

Investigation of poly(lactic acid) (PLA)/thermoplastic starch (TPS)/graphene nanoplatelets (GNP) blends revealed that the addition of GNP increased the crystallinity of the PLA/TPS blend, and the maximum crystallinity (68.39%) was observed with PLA (70%)/TPS (30%)/GNP (1%). Further increases in GNP resulted in the reduction of compatibility [81].

## 6. Applications of Biodegradable Starch-Based Nanocomposites

**Figure 9:**
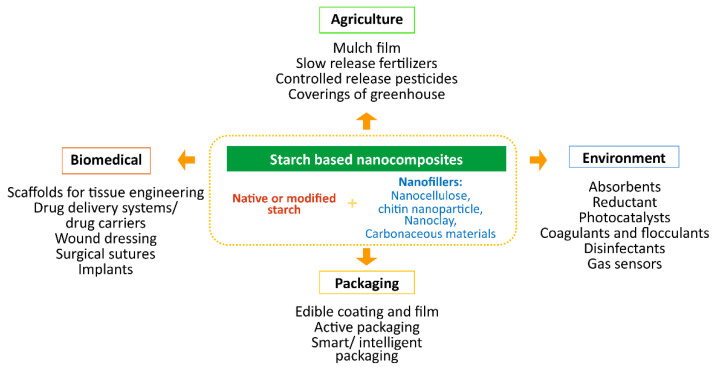
Applications of starch-based nanocomposites.

Biodegradable starch-based nanocomposites have been used in agriculture, packaging, biomedical, environment, and many other fields (Figure 9).

### 6.1. Agriculture

In recent years, biodegradable films have been developed for agricultural purposes, particularly for mulching applications, the coverings of a greenhouse, and the controlled/slow release of agrochemicals such as fertilizers and pesticides [82,83,84].

Agricultural mulches are used to prevent the hindrance caused by the weeds’ growth, maintain soil wetness, and regulate soil temperature [85]. Interaction with water (water vapor permeability, contact angle, and water solubility/resistance) and environmental factors (thermal stability) are important parameters in mulch films. Mulch films must have a very low water vapor permeability to maintain the soil moisture by reducing the water loss by evaporation. Since mulch films are exposed to outdoor conditions, improving the thermal stability is therefore essential [83,86].

Pesticides protect the crop from pests, pathogens, weeds, and insects by destroying, attacking, mitigating, or repelling activity, whereas fertilizers are essential in agriculture to increase crop yield. However, in conventional applications, the efficiency of reaching their target sites is relatively low as they are hindered by immobilization, erosion, volatilization, leaching, surface runoff, or scavenging by soil. In addition, water is also an essential factor in crop growth and driving off fertilizers. Therefore, management of nutrient/pesticide active compounds and water loss is essential for crop production. To reduce the loss and improve their utilization efficiency, slow-release fertilizers or controlled-release pesticides with improved water retention and water holding capacity can be formulated by incorporating nanomaterial into biopolymers [82,84,87,88].

Merino et al. [83] investigated the water and light interaction with corn starch-based mulch film. The study revealed that the addition of chitosan/bentonite nanofiller into native and oxidized thermoplastic corn starch improved the water resistance, radiometric, and antibacterial properties without having a significant effect on the water vapor permeation and mechanical properties [83]. In another study, Merino et al. [86] reported that the addition of bentonite/chitosan into both matrixes, native and oxidized thermoplastic corn starch, increased the crystallinity (3.0 and 3.4%) and slightly increased thermal stability in comparison to the addition of natural bentonite.

Superabsorbent hydrogels are widely used in bi-functional (retain and supply water and nutrient over a long period) slow-release fertilizers due to their water retention properties. The addition of natural char nanoparticles (NCNPs) into corn starch-g-poly(AA-co-AAm) encapsulated urea provided high biodegradability and improved the soil water-retention capacity along with the slow release of urea [84]. Chitosan (CS)/sago starch (ST)/nano zeolite (NZ) nanocomposite released 64% of phosphorus and 41.93% of urea after 14 days and increased the water retention capacity. Furthermore, CS/ST/NZ nanocomposites showed better growth indexes in *Philodendron* spp. compared to the direct application of urea, suggesting the efficacy of nanocomposites in slow-release fertilizer formulation [88]. Urea encapsulated with starch (10%)/PVA (5%) with crosslinker acrylic acid (2%) and citric acid (2%) showed higher nitrogen-releasing efficiency, 70.10 and 50.74%, respectively, as well as improved growth factors in spinach plants [89]. Modified starch (esterified with dicarboxylic acid chloride)/organobentonite-based composites regulate the effective controlled release of encapsulated pesticide atrazine [90].

### 6.2. Packaging

Food packaging protects food from humidity, high/low temperatures, and other physiological factors and aids in food quality monitoring and control in the food supply chain and during storage (gas sensors, electronic nose) [91]. Starch has been used in food packaging applications because of its strong mechanical properties, transparent/translucent appearance, and tasteless and flavorless characteristics [69]. Brittleness and poor water vapor barrier properties limit their applications. Nanoparticle reinforcement can improve the mechanical properties, hydrophobicity, water vapor and oxygen barrier, UV barrier, thermal properties, and other functional properties (antioxidant, antimicrobial, etc.) of starch which makes nanoparticles a potent material for edible film/coating, active and intelligent/smart packaging for protecting or maintaining and monitoring the quality of food materials [91,92,93].

Organic or inorganic nanofillers have been widely studied for food packaging applications, whereas organic nanofillers include nanoclay (MMTs, HNTs), natural biopolymers (chitosan, cellulose), and natural antimicrobial agents (nisin), and inorganic nanofillers includes metals (Ag, Au, Cu), and metal oxides (ZnO, TiO_2_, Ag_2_O, MgO, CuO, SnO_2_) [44,52,91].

The suitability of a film for packaging materials is mainly assessed by water vapor and oxygen barrier properties and good heat salability [94]. Furthermore, a film with improved mechanical strength and flexibility protects against shock and other physical damage. TiO_2_ NPs reinforcement in potato starch-based composite films led to a reduction in water solubility, moisture uptake, and water vapor permeability, and an increment of UV barrier properties and tensile strength of the film, showing its potential for food packaging [92]. Na-MMT and TiO_2_ NPs reduce the hydrophilicity and improve mechanical, water vapor, and UV barrier properties in cross-linked wheat starch, which makes them a suitable material for food packaging [69]. UV barrier packaging film from starch/kefiran/ZnO NPs showed improved tensile strength, Young’s modulus, and thermal stability (increased melting temperature), which are beneficial to the packaging system [95]. Starch NPs/Ag NPs showed increased antibacterial activity against *Staphylococcus aureus*, *Salmonella typhi*, and *Escherichia coli* and can be used as an antibacterial food coating material [50]. Linseed polyol increased the contact angle, water absorption capacity, thermal stability, and biodegradation of polyvinyl alcohol/corn starch film. Further addition of Ag NPs showed antimicrobial behavior against *Proteus mirabilis*, *Candida albicans*, *Escherichia coli*, *Enterococcus faecalis*, *Staphylococcus aureus*, *Klebsiella pneumoniae*, among others, which shows the potential applications in antimicrobial packaging [96]. Poly(ethyl methacrylate)-co-starch (PEMA-co-starch)/graphene oxide/Ag NPs (2 wt.%) nanocomposite film showed improved thermal stability, chemical resistance, tensile strength, oxygen barrier properties, and antimicrobial properties against *Escherichia coli*, *Pseudomonas aeruginosa*, *Staphylococcus aureus*, and *Bacillus subtilis* [97].

Plasticised corn starch films reinforced with nanocellulose improved the mechanical strength, flexibility, and water vapor and oxygen barrier properties that have a beneficial effect on reducing the oxidation of oil during storage. This film showed good heat salability, which further prevents oxygen and water vapor transmission. Moreover, the storage study ensures that this plasticized corn starch-based nanocomposite can be used as an alternative packaging material for storing edible oils at ambient conditions (27 ± 3 °C temperature, 65 ± 5% RH) for more than three months without affecting the oil quality in terms of rancidity, viscosity, and color [94].

Starch from potato, wheat, and corn blended with carboxyl methylcellulose (CMC)/Na-MMT has potential applications in food packaging [98]. Cellulose nanocrystals (CNC) obtained from sugarcane bagasse blending with starch improved mechanical, water resistance, and water barrier properties and decreased surface hydrophilicity (contact angle > 90°), which makes this starch/CNC nanocomposite a potential food packaging material [60]. Heidari et al. [61] developed edible food packaging using chitin nanofibers (CNF)/TPS nanocomposite.

TPS/MMT/carvacrol essential oil showed biocidal effects against *Escherichia coli* due to the synergistic antibacterial effect of carvacrol essential oil and MMT suggesting the applications in antimicrobial packaging [99]. Packaging material fabricated with sweet potato starch (SPS)/MMT/thyme essential oil (TEO) was studied by Issa et al. [100]. They reported that the addition of MMT improved the mechanical and water barrier properties of SPS, whereas biodegradability decreased. However, incorporating TEO decreased the tensile strength, elongation, Young’s modulus, and water barrier with improved biodegradability in SPS/MMT. The nanocomposite made from cassava starch/glycerol (2%)/Na-bentonite (0.75%)/cinnamon essential oil (2.5%) exhibited antibacterial activity against *Escherichia coli*, *Salmonella typhimurium,* and *Staphylococcus aureus*, and significantly inhibited the microbial growth in pork meatballs stored under ambient and refrigeration conditions [72].

The addition of potassium sorbate, a commonly used preservative, into starch/nanoclay films controlled the migration of sorbate, resulting in the retention of antimicrobial activity for a long period [101]. Chen et al. [102] also developed a controlled-release active film from starch/polyvinyl alcohol (PVA) incorporated with cinnamaldehyde and microfibrillated cellulose (MFC). The addition of MFC was found to improve the tensile strength, crystallinity, hydrophobicity, and antimicrobial activity (against *S. putrefaciens*) with reduced flexibility. The oxygen and water vapor permeability reduced at 1.0 and 2.5% MFC but increased at higher concentrations. In addition, MFC, at 1 and 7.5%, controlled the release of cinnamaldehyde.

Smart packaging materials for monitoring the spoilage of milk packed in a bottle were developed by incorporating pH indicators, including bromocresol green (BG) and methyl orange (MO), into a starch/nanoclay nanocomposite [93]. Further nanometals (TiO_2_, SnO_2_, Ag_2_O, MgO, ZnO, CuO) can be used in gas sensors to monitor food quality [91].

### 6.3. Biomedical

Biodegradable polymers, including starch-based bionanocomposites, are widely used as scaffolds for tissue engineering, drug delivery systems/drug carriers, wound dressing, surgical sutures, and implants due to their mechanical properties, biocompatibility, biodegradability, and also the generation of non-toxic, biodegradable products [103,104,105].

Biopolymers in the repair of healing tissues accelerate treatment processes and eliminate implant removal surgery. Furthermore, implant materials and their biodegradable products must be non-cytotoxic and biocompatible [105]. Incorporating bioactive beta-tricalcium phosphate (β-TCP) nanoparticles (at 10%) into thermoplastic starch (TPS) drastically improved the mechanical properties and showed excellent biocompatibility with no cytotoxic effect for bone tissue engineering materials [105]. Waghmare et al. [106] fabricated starch-based nanofibrous scaffolds by electrospinning for wound healing applications.

Hydroxyapatite has been used widely in biomedical applications due to its biocompatibility and osteoconductive (cell regeneration process) properties. However, brittleness and lack of flexibility limit the applications. The combination of hydroxyapatite with starch materials can reduce brittleness, and the polar nature of starch encourages a good adhesion between starch and hydroxyapatite. Sadjadi et al. [107] synthesized a nanocomposite from starch/nano-hydroxyapatite, which possesses mechanical and biological properties identical to natural bone.

Abdel-Halim and Al-Deyab [108] reported that Ag NPs/starch/polyacrylamide nanocomposite hydrogel showed antimicrobial activity against fungi (*Aspergillus flavus* and *Candida albicans*) and bacteria (*Staphylococcus aureus* and *Escherichia coli*). PVA/starch incorporated with Ag NPs synthesized from green methods (*Diospyros lotus* fruit extract) has the potential to be used in wound dressing as it shows increased swelling and moisture retention capacity and reduced water vapor transmission that prevents the wound from dehydration and better antimicrobial activity against *Escherichia coli* and *Staphylococcus aureus* [109].

The ternary blend was developed by mixing polylactic acid (PLA)/starch (S)/poly-ε-caprolactone (PCL) with nano-hydroxyapatite (nHAp) for controlled release of antibacterial triclosan. The incorporation of nHA (3%) improved the hydrolytic hydrophilicity, hydrolytic degradation, antibacterial activity (against *Escherichia coli* and *Staphylococcus aureus*), and drug release of PLA/S/PCL film. An increase in nHA content (1–7%) improved the biodegradation (13–10 months), and the antibacterial triclosan release rate of PLA/S/PCL/nHA film at 37 °C in buffer solution was increased (0.12–0.18 μg/mL every day), which is in the range of MIC of triclosan (0.025–1 μg/mL). Furthermore, the degradation and release time of PLA/S/PCL/nHA (3 wt.%) nanocomposite showed similar profiles that ensure continuous drug release during the application [110]. Mallakpour and khodadadzadeh [111] also developed starch/MWCNT modified with glucose (MWCNT-G) nanocomposites for slow release of zolpidem drug delivery. Gao et al. [112] developed spherical core-shell Ag/starch NPs using green synthesis for slow-released nano silver as an antibacterial material which can be used in pharmaceutical and biomedical applications.

Nezami et al. [113] fabricated pH-sensitive magnetic nanocomposite hydrogel using graft copolymerization of itaconic acid (IA) and starch in the presence of magnetic Fe_3_O_4_ NPs (St-IA/Fe_3_O_4_) for the controlled-release of guaifenesin (GFN) with low cytotoxicity. A nanocomposite with magnetic Fe_3_O_4_ NPs at 0.83% significantly enhanced the drug release from 54.1 to 90.4% within 24 h in pH 7.4 [113].

Starch-based-fluorescent organic nanoparticles (FONs) reported high water dispersibility and excellent biocompatibility (cell viability was 99.69% at the concentration of FONs 100 µg/mL after 24 h). Thus, FONs are a promising candidate for biomedical applications that can be potentially used as fluorescence probes and carriers for delivering biologically active components [114].

### 6.4. Environment

Extensive agricultural and industrial practices lead to the accumulation of various contaminants, including heavy metals and metalloids (Cr^6+^, Hg^2+^, Zn^2+^, Pb^2+^, Co^2+^, Cd^2+^, Cu^2+^, etc.), dyes, organic substances (pesticides, herbicides, fertilizers, aliphatic and aromatic hydrocarbons, volatile organic compounds [VOCs], oil spills), pathogenic microbes (virus, bacteria, fungi), and toxic gases (nitrogen oxides, SO_2_, CO) in water, soil, and air [115].

Starch-based nanocomposites with various nanofillers, including metal (Ag, Au, and Pd NPs), bimetal (Ag/Au), metal oxides (TiO_2_, ZnO, Fe_2_O_3_, MnO_2_), nanoscale zero-valent iron (nZVI) (Fe⁰), carbonaceous materials (CNTs [SWCNTs and MWCNTs], graphene, graphene oxide), nanoclays (MMTs, HNTs, bentonite), and polymers (chitin, cellulose nanowhiskers) are used in materials as recyclable and reusable filters, absorbents, reductants, photocatalysts, coagulants and flocculants, disinfectants, and gas sensors to detect or remediate contaminants, such as dyes, heavy metals ions (As, Pb^2+^, Cr^6+^, Cu^2+^, Cd^2+^, Hg^2+^, Ni^2+^, Co^2+^, etc.), various aromatic derivatives, fertilizers (urea), and other organic pollutants [116,117,118,119,120,121,122,123,124].

Green synthesis of Ag/Au bimetallic nanocomposite using graft copolymer hydroxyethyl starch-g-poly(acrylamide-co-acrylic acid) reported catalytic activities that involve the reduction of 4-nitrophenol to 4-aminophenol and degradation of azo dyes (congo red, Sudan-1, and methyl orange) by cleavage of −N = N-bond thus can be used in water treatment [122]. Gomes et al. [125] analyzed a starch/cellulose nanowhiskers hydrogel composite and highlighted the outstanding capacity for methylene blue dye removal.

Starch-graft-poly(acrylamide) (PAM)/graphene oxide (GO)/hydroxyapatite NPs (nHAp) nanocomposite was developed as a recyclable adsorbent for efficient removal of malachite green (MG) and other cationic dye from aqueous solution. The introduction of nHAp improved the biocompatibility of the PAM/GO composite, whereas the biodegradability, porosity, water content, and water uptake decreased with increasing nHAp content. Adsorption capacity increased with agitation time, pH, nHAp content, and initial dye concentration, and the optimum conditions were 60 min, pH 10, 5% nHAp, and 100 mg/L. PAM/GO and nHAp at 1–5 wt.% reported excellent porosity (31–11%), degradability (41–11% after 15 days), the maximum adsorption capacity of 297 mg/g, excellent regeneration capacity after five consecutive adsorption-desorption cycles of dye with high removal efficiency (77–86%) [126].

Adsorption is a basic principle of mechanism in targeted drug delivery, controlled release of pharmaceutically active compounds, and treatment of chemical water pollution [11]. The degree of the time dependency of kinetic coefficient (k_obs_) and the influencing factors (pH, temperature, initial concentration of tetracycline) are important to explore the suitability of materials in adsorption-based applications. Monodispersed starch stabilized magnetite nanoparticle (MSM) showed 70% absorption of antibiotic tetracycline within the first 5 min and reached 90% after 1 h. The degree of the time dependency of the kinetic coefficient (k_obs_) had a negative correlation with the initial tetracycline concentration [11].

Chitin nanowhiskers (CNW) are better nano-adsorbents due to their high surface/volume ratio and abundant hydroxyl and acetamide functional groups on the surface [63]. MMT is hydrophilic and has a high specific area [127]. The bean starch/Na-MMT nanocomposite showed high absorption capacity for heavy metals Ni^2+^ (97.1% at pH 4.5, initial concentration of 100 ppm) and Co^2+^ (78.03% at pH 6, initial concentration of 140) in comparison to the starch matrix (72 and 74.2%, respectively) [116]. Yang et al. [123] studied the material nZVI loaded on biochar stabilized by starch to remediate Cr^6+^.

Enzyme immobilization is an emerging technology for environmental remediation which gives many advantages over free enzymes, which include the efficiency and stability of catalytic enzymes and their enhanced recovery and reusability [128]. Further, the immobilized enzyme can be used as biosensors and biocatalysts to degrade dye from textile, leather, coloring, and printing industries [129]. Immobilized peroxidase on polymer/Fe_3_O_4_ magnetic NPs has been successfully used to remediate wastewater containing different dyes in the textile industry [128]. Immobilized phenoloxidases other than peroxidase, including laccase and tyrosinase, are also used to degrade dyes and phenolic pollutants, and lipases are used to remediate oily wastewater [130]. Mehde [131] reported that magnetic NPs/tannic acid/starch/cross-linked enzyme aggregates-peroxidase are used to remove different types of dyes, such as methylene blue, Congo red, indigo carmine, and malachite green.

### 6.5. Other Applications

Plasticized starch/reduced graphene oxide (rGO) nanocomposites with improved conductivity and dielectric properties can be used in bio-friendly flexible electronic devices [80]. The maize starch/glycerol (20%)/Na-MMT (10%) nanocomposite showed improved tensile properties, which can be used in lightweight architectural constructions [66]. Starch-based nanocomposites can also be used in lithium batteries, fuel cells, dye-sensitized solar cells, and electrically conductive biocomposite film for various other purposes [22,77].

Table 2 summarizes the studies reported on various biodegradable starch-based nanocomposites in regard to their applications and properties.

## 7. Lifecycle Analysis of Nanocomposites

With increasing fossil depletion and environmental concerns, sustainable biobased materials have gained increasing interest. For biobased materials to be sustainable, preparation and processing should have limited environmental impacts [132].

The environmental credentials of bionanocomposites are evaluated by assessing their material production, product manufacturing, and product end-of-life. Many tools, including environmental impact analysis (EIA), life cycle analysis (LCA), material flow analysis (MFA), and ecological footprint (EF), are used for analyzing the environmental impacts of materials and manufacturing processes [133]. Life cycle assessment (LCA) is the most widely accepted method to assess environmental impact [134]. LCA is a science-based tool to comparatively analyze the environmental impacts of product systems concerning the extraction of raw materials, manufacturing, the use of final products, and their disposal [133,135,136].

**Figure 10:**
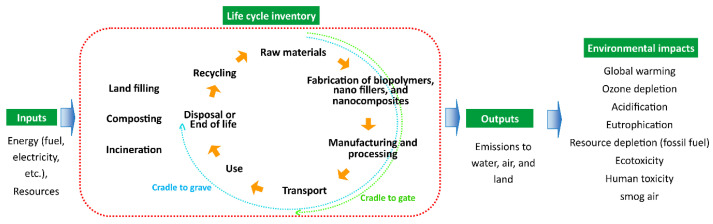
A general framework for the LCA of nanocomposite materials.

The international organization for standardization (ISO) standardized the LCA via ISO 14040 series [134]. The two most commonly used methods are “*cradle to grave*” and “*cradle to gate*” [134,135]. The “*cradle to gate*” system covers all the steps from raw material extraction and energy to product conversion and delivery at the factory gate, whereas “*cradle to grave*” covers all phases of the lifecycle of a product, i.e., includes all steps of “*cradle to gate*” and usage and disposal phase [134]. LCA can be investigated through several environmental impact categories, such as global warming, ozone depletion, acidification, eutrophication, resource depletion (fossil fuel), ecotoxicity, human toxicity, photo oxidant formation, smog air, etc. [136,137,138]. Thus it is difficult to compare the results between studies [138]. Furthermore, there are only very few mentions in the literature about the environmental performance of nanomaterials based on LCA methods which also has some limitations, including a lack of life cycle inventory data and characterization factors for NMs’ emissions [139,140]. Figure 10 depicts the simplified framework for the LCA of nanocomposite materials.

This section covers the environmental profile of starch-based nanocomposites in comparison to nonconventional counterparts. The environmental impacts of starch-based composites production with PBS, PLA/PBAT, PHB, PLA, PBS/fiber, and recycled-PLA were greatly varied: non-renewable energy use (NREU) (33–72 MJ/kg, when using virgin starch), eutrophication (1.2–1.9 g P eq./kg), greenhouse gas (GHG) emissions (1.8–3.7 kg CO_2_ eq./kg) and agricultural land use (0.3–1.3 m^2^yr/kg) (Table 3). Compared to petrochemical polymers, LDPE and PP, virgin starch-based polymers reduced GHG emissions (up to 80%, except starch/PBS, starch/PLA/PBAT) and NREU (up to 60%) but increased eutrophication potential (up to 400%) and agricultural land use. Furthermore, reclaimed starch from wastewater instead of virgin starch reduced environmental impacts [141].

The microwave-assisted technique can be an environmentally friendly alternative for glucose-reduced and starch-stabilized Ag NPs production [137].

LeCorre et al. [132] compared the sustainability of extraction of nanofillers’ starch nanocrystals (SNC) and organically modified nanoclay montmorillonite (OMMT). Though global warming and acidification potential indicators of SNC were higher than those of OMMT, SNC has more positive impacts than OMMT, which contributes to non-renewable energy and mineral depletion.

The choice of starch sources and plasticizers influences the environmental impacts displayed by the production of composites. Corn starch/glycerol exhibited the lowest impact on the ecosystem, human health, and resources [142].

**Table 3:** Environmental impacts of starch polymer and nanofiller compared with LDPE polymer (Functional unit = 1 kg).

Impact Category	Ozone Depletion (kg CFC-11 eq.)	Global Warming Potential/Greenhouse Gas Emissions (kg CO_2_ eq.)	Smog (kg O_3_ eq.)	Acidification (kg SO_2_ eq.)	Eutrophication (kg N eq.)	Human Toxicity, Carcinogen (CTUh)	Human Toxicity, Noncarcinogen (CTUh)	Respiratory Effects (kg PM2.5 eq.)	Ecotoxicity (CTUe)	Water Consumption (Kg)	Agricultural Land Use (m^2^yr/kg)	Fossil Fuel Depletion/Non-Renewable Energy Use (MJ Surplus)	Reference
PE waste management	1.28 × 10^−5^	3.82 × 10^3^		5.77 × 10	1.39 × 10 ^c^							1.05 × 10^−4^	[143]
Starch-based polymers production with PBS, PLA/PBAT, PHB, PLA, PBS/fiber, and recycled-PLA		1.8–3.7			1.2–1.9 ^d^						0.3–1.3	33–72	[141]
Starch-stabilized Ag NPs manufacturing via microwave-assisted heating	1.24 × 10^−7^	8.44 × 10^−2^	2.37 × 10^−1^	2.51 × 10^−1^	1.21 × 10^−1^	4.44 × 10^−6^	8.02 × 10^−4^		6.41 × 10^2^	5.85 × 10^5^		7.08 × 10^2^	[137]
Starch nanofiller preparation using various process	0.00	7.95–13.07	0.5–0.6 ^a^	8.78–15.51 ^b^	0.16–0.23	0.9–0.16 ^e^	2216.99–3747.76 ^f^	0.02	33.15–115.82 ^h^			16–19	[132]
Nanofiller OMMT		1.52						1.139 ^g^				40.079	[144]

^a^, kg/NOx eq.; ^b^, H^+^ moles eq.; ^c^, kg PO_4_^3-^ eq.; ^d^, g P eq./kg; ^e^, kg benzene eq.; ^f^, kg toluene eq.; ^g^, g PM; ^h^, kg 2,4-D eq. PM2.5, particulate matter of size under 2.5 µm; 2,4-D, 2,4-dichlorophenoxyacetic acid used as a herbicide and pesticide.

## 8. Biodegradation of Starch

Based on ASTM, biodegradable is defined as ‘capable of undergoing decomposition into carbon dioxide, methane, water, inorganic compounds, or biomass in which the predominant mechanism is the enzymatic action of microorganisms that can be measured by standard tests, in a specified period, reflecting available disposal condition’ [44]. Biodegradable polymers play a critical role in environmental sustainability as they take part in the natural cycle “from nature to nature” [145]. With regard to biopolymer, to be certified as a biodegradable material, 90% of its mass should be decomposed in composting conditions within 90 days [146].

The type, nature, concentration, chemical modification, and antimicrobial properties of nanofiller, biodegradation test methods, and parameters, including temperature, moisture, humidity, pH, quantity and type of microorganisms, etc., can influence the biodegradability of nanocomposites [15,145,147].

Starch modification and incorporation of nanomaterials as nanofiller have been shown to alter biodegradability. For example, the biodegradability of starch increased with the addition of MMT at lower concentrations because of increased hydrophilicity that permits the microorganisms to enter into the polymer. In contrast, chemical modification of starch, nanofillers such as TiO_2_, graphene oxide, etc., reduce the biodegradability of starch-based nanocomposite because of their antioxidant potential [5,14,15,148].

Crosslinked nanocomposite film produced from thermoplastic corn starch crosslinked with oxidized sucrose and reinforced with cellulose nanofibrils from a pineapple leaf was reported to have a 30% weight loss rate after 30 days of burial, much lower than that of thermoplastic starch (80%) [5]. Crosslinking thermoplastic starch is hard to decompose due to the formation of acetal/hemiacetals and reduction of hydrophilicity and water permeability of nanocomposite, which decrease the attraction and permeability of microorganisms into the polymer matrix [5].

The addition of MMT into sweet potato starch (SPS) hindered biodegradability in soil burial tests due to the strong hydrogen bond between the hydroxyl groups of SPS and MMT and decreased water solubility that prevents water diffusion into the film [100]. However, the effect of MMT on biodegradability is concentration dependent. In corn starch-based film, adding MMT nanoclay at a lower concentration (1–3%) delayed the biodegradation rate (22–23 days for complete degradation), which may be attributed to the formation of the exfoliated structure at a lower concentration of MMT, which ensures good interaction between MMT and the polymer matrix. The biodegradability was increased at a higher level (>3%) of MMT due to agglomeration [15].

The cationic starch-based film incorporated with MMT and nanocrystalline cellulose degrade faster than the pure cationic starch film in composting at 58 °C, which may be attributed to hydrophilic nanocrystalline cellulose [127]. Thyme essential oil (TEO) and MMT incorporation have also been shown to increase biodegradation in SPS/MMT nanocomposites [100].

Incorporating fibrous TiO_2_ (0.01 and 0.05 wt.%) in maize starch/PVA composite films improved the tensile strength, water vapor, and UV barrier properties with little effect on biodegradability in soil [146]. The addition of nanoclay fillers delays the biodegradation of corn starch when buried in a microbiological medium of pure *Micrococcus luteus* culture at room temperature for 30 days [15]. Incorporating antimicrobial Ag NPs into starch/PVA composite film reduced its biodegradability [14].

The addition of CaCO_3_ in starch/polyethylhexylacrylate (PEHA)/PVA composite film improved the tensile strength, thermal stability, chemical resistance, and antimicrobial properties, which can be suitable for packaging. Starch/PEHA/PVA/CaCO_3_ degraded by 65% after 15 days in activated sludge water [149]. Food packaging material prepared from poly(ethyl methacrylate)-co-starch/graphene oxide/AgNPs showed only a 4.5% biodegradation in active sludge water after 180 days [97].

Poly(lactic acid) (PLA)/thermoplastic cassava starch (TPCS)/graphene nanoplatelets (GRH) nanocomposite film showed a lower biodegradation rate than PLA film in vermiculite (0.11 to 0.06 d^−1^) and compost media (0.09 to 0.08 d^−1^) [148].

In slow-release fertilizer formulation, the incorporation of natural char nanoparticles (NCNPs) into corn starch-g-poly (acrylic acid-co-acrylamide)/urea composite increased the degradation rate (23.9% after 30 days in soil), which may be attributed to the increment in water absorbance that promotes the soil microorganisms to enter into the polymer matrix [84].

The biodegradability of nanocomposite film polylactic acid/starch/poly-ε-caprolactone/nano hydroxyapatite (nHAp) was increased with the nHAp content [110]. Hosseinzadeh and Ramin [126] reported that the degradability of starch-graft-poly(acrylamide) (PAM)/graphene oxide (GO) nanocomposite decreased with increasing nHAp addition in buffer solution due to the higher crystallinity, compressive strength, and elastic modulus of nanocomposite film.

In vitro degradation tests performed in a simulated body fluid (SBF) showed that thermoplastic starch (TPS)/beta-tricalcium phosphate (β-TCP) NPs degraded 51% after 28 days, higher than that of TPS (47%) [105]. Table 4 summarizes the recent findings about the biodegradability of different starch-based biopolymers.

## 9. Conclusions and Future Perspectives

In summary, starch is a natural polymer with outstanding biocompatible characteristics and can be used as both a matrix and reinforcement material for the development of new bionanocomposites. Starch nanoparticles and other nanofillers, including nanocellulose, chitin NPs, nanoclay (MMT, HNTs, bentonites), carbon nanoparticles (MWCNTs, SWCNTs, graphene, graphene oxides), metal and metal oxides (Ag NPs, TiO_2_, ZnO, CaCO_3_, etc.), have been widely used for the creation of new starch-based bionanocomposites and are promising candidates for various industrial applications.

The excellent biocompatibility, complete degradability without toxic residues, low cost, wide availability, and renewability of starch-based nanocomposites would open up many applications in agriculture, packaging, environmental remediation, biomedicine, and many other fields. Some of the reported applications are edible food coating, active and intelligent food packaging, controlled/slow-released pesticides and fertilizers, mulch films, drug carriers (controlled/target specific), wound healing, scaffolds in tissue engineering, absorbents, filters, catalysts, or disinfectants for environmental remediation, electronic devices, lightweight architectural constructions, stabilizers in food and paints such as non-food applications, and many others.

Modification of starch or reinforcement with other materials to form a nanocomposite may alter biodegradability. Therefore, regarding the biodegradability of starch-based nanocomposites is important for them to be claimed as being biodegradable materials. Life cycle assessment of starch-based biocomposite materials for their respective applications provides critical information regarding the environmental and ecological benefits of the materials over fossil-based synthetic polymers for developing sustainable nanocomposites. However, only few studies have focused on life cycle assessment. Therefore, further studies on life cycle assessment of starch-based nanocomposites needs to be investigated. Nanomaterials can also enter the human body through inhalation, contact, and ingestion, which can lead to their accumulation in the human body, Therefore, further investigations on toxicity and risk factor analysis are necessary to find the most suitable starch-based nanocomposite materials.

## Figures and Tables

**Table 1:** Amylose and amylopectin contents of starch from various sources.

Starch Source	Amylose (%)	Reference
Arrowroot	35.52	[27]
Banana (pulp)	16.36–26.2	[28,29,30]
Banana (peel)	25.7	[29]
Barley (regular)	24.7	[31]
Cassava	2.5–32.12	[28,32,33]
Corn	0–79.05	[28,32]
Maize (normal)	22.7–28.9	[31,34]
Maize (waxy)	0.18	[34]
Maize (high amylose content)	35.5–64.8	[34]
Potato	18.6–31.9	[28,31,32,33]
Rice	0.1–28.7	[20,35]
Sweet potato (normal)	30.4	[36]
Wheat	6.2–22.8	[31,32]

**Table 2:** Starch-based nanocomposites using various biodegradable polymers in regard to their applications and properties.

Starch-Based Nanocomposites	Application	Properties	References
Native (TPS) or oxidized (TPS-ox) corn starch/chitosan (CS)/bentonite (Bent)	Mulch film	The addition of 4% CS/Bent improved water resistance (decreased water solubility), radiometric, and antibacterial properties. Decreased mechanical property (tensile strength and elastic modulus: TPS-ox > TPS-ox/CS/Bent > TPS > TPS/CS/Bent).	[83]
Native (TPS) or oxidized (TPS-ox) corn starch/chitosan (CS)/bentonite (Bent)	Mulch film	The addition of 4% CS/Bent increased the crystallinity (3.30 and 3.00%) and led to a slight increase in thermal stability (T_max_ 139.2 and 126.9 °C) in TPS and TPS-ox, respectively.	[86]
Corn starch-g-poly(AA-co-AAm)/natural char nanoparticles (NCNPs)/urea	Bi-functional slow-release fertilizers	Provided improved biodegradability, soil water-retention capacity (35.6% and 33.2% at pH 4.5 and 5.5, respectively, after 6 days), water absorbency (215.1 g/g) along with the slow release of urea (73% in deionized water and 37% in NaCl).	[84]
Urea encapsulated with starch (10%)/PVA (5%) with crosslinker acrylic acid (2%) and citric acid (2%)	Slow release of fertilizer	Releasing efficiency of starch/PVA/acrylic acid and starch/PVA/citric acid were 70.10 and 50.74%, respectively. Improved the growth factors in spinach plants	[89]
Corn starch/Debranched starch NPs (DSNPs)	Food packaging	Addition of 5% DSNPs increased the tensile strength (from 0.95 to 1.73 MPa) and decreased the water vapor permeability (7.11 to 4.91 × 10^−^^10^ gPa^−1^h^−1^m^−1^) and oxygen transmission rate (394 to 81.61 cm^3^/m^2^⋅day)	[49]
Starch NPs/Ag NPs	Coating material for food packaging	Antibacterial activity against *Staphylococcus aureus*, *Salmonella typhi*, and *Escherichia coli*.	[50]
Cross-linked wheat starch (CLWS)/sodium montmorillonite (Na-MMT)/TiO_2_ NPs	Food packaging material	Showed exfoliated structure. Adding Na-MMT (5%) and TiO_2_ NPs (1%) into CLWS showed reduced water vapor permeability (from 9.1 to 4.8 × 10^−5^ g/m.d.Pa) and water solubility (100–50.35%), and increased thermal stability, tensile strength (2.49–5.56 MPa), and Young’s modulus (0.71–1.09 MPa) in comparison to native wheat starch. CLWS/Na-MMT/TiO_2_ NPs showed better UV-blocking properties than CLWS/Na-MMT.	[69]
Sweet potato starch (SPS)/montmorillonite (MMT)/thyme essential oil (TEO)	Food packaging	The addition of MMT improved the tensile (44.91%), Young’s modulus (135.69 MPa), and water vapor barrier (0.022 gm/m^2^/day) and hindered the biodegradability of SPS. The addition of TEO decreased the mechanical and water vapor barrier properties of SPS/MMT nanocomposites. The addition of MMT and TEO improved water resistance by 50%.	[100]
Starch (potato, wheat, and corn, high amylose corn) carboxyl methylcellulose (CMC)/Na-MMT	Food packaging	Corn starch/CMC/Na-MMT nanocomposite showed higher tensile strength, glass transition temperature, thermal stability, crystallinity, lower solubility, and water vapor permeability.	[98]
Cassava starch/glycerol/Na-bentonite nanoclay/cinnamon essential oil	Antimicrobial food packaging pork meatballs	Antibacterial activity against *Escherichia coli*, *Salmonella typhimurium,* and *Staphylococcus aureus*. Improved the antimicrobial efficacy in pork meatballs stored under ambient and refrigeration conditions.	[72]
Starch/polyvinyl alcohol (PVA)/cinnamaldehyde (Cin)/micro fibrillated cellulose (MFC)	Controlled-release active packaging film	MFC improved the tensile strength, crystallinity, hydrophobicity, and antimicrobial activity (against *S. putrefaciens*) with reduced flexibility. The oxygen and water vapor permeability reduced at 1 and 2.5% MFC and increased at higher concentrations. MFC at 1 and 7.5% controlled the release of Cin.	[102]
Corn starch (CS)/nanocellulose (NC)/glycerol (GL)/polyvinyl alcohol (PVOH)	Packaging material for edible oil	Optimum composition for CS-based nanocomposite: 0.89% NC, 2.53% GL, and 1.89% PVOH. Tensile strength 8.92 MPa, elongation at break 41.92%, bursting strength 556 kPa, and WVP 7.07 × 10^−10^ g/m.s.Pa, oxygen transmission rate 3.56 × 10^−5^ cm^3^/m^2^ d.Pa. Good heat salability.	[94]
Starch from unripe plantain bananas/cellulose nanofibers from banana peels	Food packaging	Homogenized nanocomposite at five times higher pressure increased the tensile strength (from 7.3–9.9 MP), Young’s modulus (478.6–663.1 MPa), decreased the elongation at break (32.2–20.7%), solubility (32.3–29.0%), WVP (10.7–6.0 × 10^−11^ g/m.s.Pa at low RH), sorption (2.73–2.20 × 10^−7^ mm^2^/s), and diffusion coefficient (0.42–0.27).	[59]
Corn starch (CS)/nanocellulose fiber (NCF)/thymol	Antioxidant and antimicrobial food packaging	Adding 1.5% of NCF improved the thermal stability, mechanical and water vapor, and oxygen barrier properties of corn starch film. CS/NCF/thymol composite reported improved thermal stability and flexibility with decreased tensile strength, Young’s modulus, and barrier properties.	[58]
Starch/cellulose nanocrystals (CNC)	Food packaging	Improved the tensile strength (2.8 to 17.4 MPa), Young’s modulus (112 to 520 MPa), water resistance (reduced solubility 26.6 to 18.5%), and water barrier properties and decreased surface hydrophilicity (contact angle 38.2 to 96.3°).	[60]
TPS/chitin nanofibers (CNF) from fungus *Mucor indicus*	Nanocomposite for food packaging and other applications.	Addition of 5 wt.% CNF enhanced Young’s modulus (239%) and tensile strength (by 180%) and reduced the elongation at break and moisture absorption compared to the TPS film.	[39]
PVA/starch/Ag NPs from *Diospyros lotus* fruit extract	Wound dressing applications	Increased swelling and moisture retention capacity, reduced water vapor transmission. Better antimicrobial activity against *Escherichia* *coli* and *Staphylococcus aureus*	[109]
Thermoplastic starch (TPS)/beta-tricalcium phosphate (β-TCP) NPs	Bone tissue engineering materials	Adding β-TCP at 10% improved the tensile strength (from 1.67 to 4.8 MPa) and Young’s modulus (from 66.54 to 390.5 MPa), and decreased elongation at break (78.56 to 18.03%) of TPS. Exhibited non-cytotoxicity effects and excellent biocompatibility.	[105]
Polylactic acid (PLA)/starch (S)/poly-ε-caprolactone (PCL)/nano hydroxyapatite (nHAp)/	Controlled release of antibacterial triclosan	Incorporating nHA (3%) improved the hydrolytic hydrophilicity, hydrolytic degradation, antibacterial activity (against *Escherichia* *coli* and *Staphylococcus* *aureus*), and continuous drug release of PLA/S/PCL film.	[110]
Starch-itaconic acid/Fe_3_O_4_ NPs (St-IA/Fe_3_O_4_)	Controlled release of Guaifenesin (GFN)	The addition of magnetic Fe_3_O_4_ NPs at 0.83% enhanced the drug release percentage from 54.1 to 90.4% within 24 h in pH 7.4. Adding Fe_3_O_4_ NPs improved the wound healing ability in mice (healed after 10 days). Exhibited low cytotoxicity for human umbilical vein endothelial cells.	[113]
Graft copolymer hydroxyethyl starch-g-poly(acrylamide-co-acrylic acid)/Ag-Au bimetallic nanocomposite	Removal of toxic azo dyes from wastewater	Catalytic activities: reduction of 4-nitrophenol to 4-aminophenol and degradation by cleavage of −N = N-the bond of azo dyes (Congo red, Sudan-1, and methyl orange).	[122]
Starch-graft-poly(acrylamide) (PAM)/graphene oxide (GO)/hydroxyapatite NPs (nHAp) nanocomposite	Recyclable adsorbent for efficient removal of malachite green (MG) dye from aqueous solution	PAM/GO and nHAp at 1–5 wt.% reported excellent porosity (31–11%), degradability (41–11% after 15 days), the maximum adsorption capacity of 297 mg/g, excellent regeneration capacity after five consecutive adsorption-desorption cycle of dye (27–14% of MG dye was liberated after 5th cycle, i.e., 77–86% removal efficiency)	[126].
Bean starch/sodium montmorillonite (Na-MMT)	Removal of Ni^2+^ from water	Adding Na-MMT improved the absorption yield for Ni^2+^ (from 72 to 97.1% at pH 4.5, initial concentration of 100 ppm) and Co^2+^ (74.2 to 78.03% at pH 6, initial concentration of 140) in comparison to the bean starch matrix.	[116]
MWCNT/starch plasticized with ionic liquid, 1-ethyl-3-methylimidazolium acetate ([emim+][Ac−])	Packaging, lithium batteries, fuel cells, and dye-sensitized solar cells	MWCNT at 0.5 wt.% increased the tensile strength, Young’s modulus, and elongation at the break by 327%, 2484%, and 82%, respectively. Electrical conductivity increased with MWCNT content with the maximum (56.3 S/m) at 5 wt.% MWCNT. Starch plasticizer [emim+][Ac−] slightly decreased the thermal stability in comparison to glycerol in the MWCNT/starch nanocomposite.	[77]
Starch/MWCNT/surfactants such as sodium dodecyl sulfate (SDS), cetyltrimethylammonium bromide (CTAB), and sodium cholate (SC)	Electrically conductive biocomposite film	CTAB reduced the mechanical properties of starch, while SC had no significant effect. SC (18.3–25.3°) and CTAB (20.8–32.3°) reduced the contact angle of starch (42.9–45.2°). CTAB (14.75 S/m) and SC (11.56 S/m) improved the electrical conductivity of starch (2.03 × 10^−6^ S/m). CTAB (30.2%), SDS (24.4%), and SC (12%) increased the inhibition of free radicals more than starch.	[22].
Maize starch/glycerol (20%)/Na-MMT (10%) nanoclay	Lightweight architectural constructions	Showed intercalated structure and improved tensile properties.	[66]

**Table 4:** Biodegradability of different starch-based biopolymers.

Starch-Based Nanocomposite	Method	Biodegradation	Other Observation	Reference
Poly(ethyl methacrylate)-co-starch/graphene oxide/Ag NPs (PEMA-co-starch/GO/Ag NPs)	Active sludge water for 180 days.	4.5% after 180 days.	GO and Ag NPs (2 wt.%) increased thermal stability, chemical resistance, tensile strength, and oxygen barrier property. Antimicrobial activity against *Escherichia coli*, *Pseudomonas aeruginosa*, *Staphylococcus aureus*, and *Bacillus subtilis*.	[97]
Maize starch/PVA/TiO_2_	Soil burial test: buried at 2–3 cm depth in peaty soil with 60% moisture, 98% RH, at 30 °C for 3 months.	Around <20% remaining mass after 80 days.	The addition of fibrous TiO_2_ (0.01 and 0.05 wt.%) decreased the elongation at break and improved the tensile strength, Young’s modulus UV, and water vapor barrier properties.	[146]
Sweet potato starch (SPS)/montmorillonite (MMT)/thyme essential oil (TEO)	Soil burial degradation test.	The addition of MMT hindered the biodegradability (23.25%) of SPS (48.88%). Biodegradability of SPS/MMT increased with the addition of TEO (61–63%)	The addition of MMT and TEO improved water resistance by 50%. The addition of MMT at 3% and TEO at 2% improved the elongation, Young’s modulus, and water vapor barrier properties of SPS.	[100]
Corn starch/glycerol/montmorillonite (MMT) nanoclay	Microbiological medium of pure *Micrococcus luteus* culture incubating at room temperature for 30 days.	Complete decay after 20 days in corn starch and 21–24 days in corn starch filled with nanoclay.	Addition of nanoclay (2–3 wt.%) in corn starch reduced water absorption (by 22%), moisture uptake (40%), oxygen permeation (30%), and swelling thickness (31%).	[15]
Cationic starch (CS)/montmorillonite (MMT)/nanocrystalline cellulose (NCC)	Composting conditions at 58 °C for 26 days.	CS/MMT/NCC nanocomposite films showed a higher decomposition rate than pure CS. 90% disintegration after 26 days.	Addition of MMT (5% wt) and NCC (5% wt) increased tensile strength (6.60 MPa) and modulus (2.17 GPa), and decreased elongation at break, water solubility (19.63%), moisture absorption (17.73%), water vapor permeability (4.61 gMm.m^−2^day.kPa), O_2_ permeability (28.72 cm^3^m^−1^d^−1^Pa^−1^).	[127]
Cross-linked poly(lactic acid) (PLA)/maleated thermoplastic starch (MTPS)/montmorillonite (MMT)	Samples (1.5 × 1.5 cm) in activated sludge for 3 months.	MTPS and nanoclay improved the biodegradation, while crosslinking of PLA reduced the biodegradation rate.	The addition of MMT improved tensile strength. Increasing MTPS (wt.%) content decreased the tensile strength and increased the elongation at break.	[71]
Corn starch-g-poly(AA-co-AAm)/natural char nanoparticles (NCNPs) nanocomposite encapsulated urea. Where: acrylic acid (AA), acrylamide (AAm).	Buried in the soil at pH 7.5 for 30 days.	The degradation rate after 30 days was 23.9%.	The addition of NCNPs decreased the leaching of nitrate and improved soil water-retention capacity.	[84]
Thermoplastic corn starch (TPS)/cellulose nanofibrils from pineapple leaf/oxidized sucrose	Sample (40 × 8 × 2 mm) buried at 10 cm depth of a sand and soil mixture (in equal ratio) at ambient temperature for 30 days.	About 30% weight loss in cross-linked films after 30 days, much lower than TPS (80%).	-	[5]
Starch/polyethylhexylacrylate (PEHA)/polyvinylalcohol (PVA)/nano CaCO_3_ nanocomposite	Activated sludge water for 90 days.	Starch/PEHA/PVA/CaCO_3_ (8 wt.%) degraded by 65% after 15 days	CaCO_3_ increased the tensile strength, thermal conductivity, thermal stability, and chemical resistance. Antimicrobial activity against *Candida albicans*, *Escherichia coli,* *Pseudomonas* *aeruginosa*.	[149]
Poly(lactic acid) (PLA)/thermoplastic cassava starch (TPCS)/graphene nanoplatelets (GRH)	Samples (1 cm^2^) buried in inoculated vermiculite and compost under aerobic controlled conditions: at 58 ± 2 °C, RH 50 ± 5%, and airflow rate 40 ± 2 cm^3^min^−1^).	The addition of GRH decreased the biodegradation rate from 0.11 to 0.06 d^−1^ in vermiculite and 0.09 to 0.08 d^−1^ in compost media.	In PLA, adding TPCS and GRH reduced the crystallinity (34.5 to 4.5%).	[148]
Polylactic acid (PLA)/starch (S)/poly-ε-caprolactone (PCL)/nano hydroxyapatite (nHAp)/	In-vitro hydrolytic degradation test, 0.15 g samples (1 × 1 × 0.15 cm) was hot pressed and incubated in 50 mL phosphate buffer with pH 7.4 at 37 °C.	The increase in nHAp content (1–7%), faster the degradation (13–10 months).	Incorporating nHA (3%) improved the hydrophilicity and antibacterial activity (against *Escherichia* *coli* and *Staphylococcus* *aureus*).	[110]
Starch-graft-poly(acrylamide) (PAM)/graphene oxide (GO)/hydroxyapatite NPs (nHAp) nanocomposite	Soaked in PBS buffer solutions (pH 7.4) containing lysozyme (5000 U/mL) at 37 °C for 15 days.	Biodegradation decreased with increasing nHAp content. Degradability was 41–11% lower than that of PAM/GO (55%) after 15 days.	With increasing nHAp content, porosity, water content, and water uptake were decreased.	[126]
Thermoplastic starch (TPS)/beta-tricalcium phosphate (β-TCP) NPs	In vitro degradation tests were performed in a simulated body fluid (SBF) for 28 days.	Degraded 51% after 28 days, higher than TPS (47%).	Adding β-TCP at 10% improved the mechanical properties of TPS.	[105]
Starch/PVA/Ag NPs	Under controlled aerobic composting conditions at 58 ± 2 °C for 45 days (based on EN ISO 14855-1: 2012 standard). Disintegration test under composting conditions: 5 g of film samples (25 × 25 mm) at 58 ± 2 °C for 73 days (ISO 20200: 2004).	Biodegradation is 58% after 45 days, which is higher than that of PVA (54%) and lower than starch (134%). Poor disintegration behavior in comparison to starch.	-	[14]
Polyvinyl alcohol (PVA)/corn starch (CS)/linseed polyol (LP)/Ag NPs	Soil burial of samples (2 × 2 cm) at a depth of 10 cm.	Biodegradability after 4 weeks PVA < PVA/CS < PVA/CS/LP < PVA/CS/LP/Ag NPs	Improved contact angle (53°), water absorption capacity (equilibrium swelling percentage 129%), thermal stability (10% weight loss at 308 °C), and biodegradation than PVA/CS film. Ag NPs improved antimicrobial behavior against *Proteus mirabilis*, *Candida albicans*, *Escherichia coli*, *Enterococcus faecalis*, *Staphylococcus* *aureus*, *Klebsiella pneumoniae*, among others.	[96]

## Data Availability

Not applicable.
